# Ambulophobia as a Specific Phobia—Defining the Problem Among Patients of Long-Term Care Facilities in Poland

**DOI:** 10.3389/fpubh.2022.801109

**Published:** 2022-04-12

**Authors:** Michał Górski, Marta Buczkowska, Karolina Krzywkowska, Beata Całyniuk, Karolina Górska, Krzysztof Buczkowski, Joanna Fojcik, Mateusz Grajek, Renata Polaniak

**Affiliations:** ^1^Doctoral School of the Medical University of Silesia in Katowice, Faculty of Health Sciences in Bytom, Medical University of Silesia, Katowice, Poland; ^2^Department of Toxicology and Health Protection in the Occupational Environment, Faculty of Health Sciences in Bytom, Medical University of Silesia, Katowice, Poland; ^3^Department of Psychiatric Rehabilitation in Katowice, Medical University of Silesia, Katowice, Poland; ^4^Department of Human Nutrition, Faculty of Health Sciences in Bytom, Medical University of Silesia, Katowice, Poland; ^5^Institute of Special Pedagogy, School and Teacher Education, Pedagogical University of Cracow, Cracow, Poland; ^6^Department of General and Oncological Surgery, City Hospital of Siemianowice Śla̧skie, Siemianowice Slaskie, Poland; ^7^Department of Public Health, Faculty of Health Sciences in Bytom, Medical University of Silesia, Katowice, Poland

**Keywords:** ambulophobia, specific phobia, anxiety disorders, long-term care, aging

## Abstract

**Background:**

Ambulophobia is a type of specific phobia that involves a fear of walking. This phobia mainly affects older people, who prefer not to leave their bed or home to avoid walking on uneven surfaces and reduce the risk of falling. The problem seems to be very important in terms of public health and the organization of long-term care, as Poland has one of the highest rates of elderly population growth.

**Objectives:**

The aim of the study was to determine the prevalence of ambulophobia among patients of long-term care facilities in Poland and to identify factors increasing the risk of this specific phobia in the study group.

**Material and Methods:**

The study was conducted between January and July 2021. Data collected from 379 patients of 16 long-term care facilities located in Poland were analyzed. The study used the diagnostic criteria for specific phobias according to the Diagnostic and Statistical Manual of Mental Disorders, 5th Edition, and standardized questionnaires such as MMSE, GDS-16, DOS and ADL. The χ^2^ test was used to test the significance of differences (*p* = 0.05).

**Results:**

The prevalence of ambulophobia in the study group was 30.1%. Ambulophobia significantly more often concerned the female sex (37.7%) and people over 70 years of age (42,1%). Factors such as depression, Parkinson's disease, orthostatic hypotonia, a history of falling or being a witness to another person falling, and disability of at least a moderate degree increased the likelihood of ambulophobia.

**Conclusions:**

Based on the obtained results, it was found that the protective factors in the development of ambulophobia are male sex, younger age, high independence, fewer drugs used per day and no previous falls or seeing another person fall.

## Introduction

Poland is among the leading countries in Europe with the fastest aging population (1). By 2050, the number of seniors will double up, which is associated with many health and psychological consequences (1). One of the most serious geriatric problems is falls in older people. Progressive degenerative changes with age, coexisting diseases and medications used, as well as past injuries, limiting the agility of older people, promote the occurrence of falls (2). The importance of falls is related to their consequences in the form of physical and psychological injuries (2). According to the WHO (3) definition, a fall is an event whereby a person unintentionally ends up on the floor or other low-lying surface as a result of a loss of balance, such as when walking. According to epidemiological data, the fear of falling is not unfounded. Studies show that among people over 65 years of age, they fall at least once a year: 50–67% of nursing home residents, 33% of people living independently and 20% of those hospitalized (4). According to statistics, as many as 10% of falls end in serious injuries (4). However, 1–2% of them results in a fracture of the hip fracture (4, 5). Fear of walking is much more common in older people than in children and adults. This is due to the presence of certain risk factors specific to older people, which include osteoporosis, visual impairment, blindness or balance disorders, dizziness, joint dysfunction, syncope due to circulatory disorders, and as a result of medication (6). Risk factors also include multimorbidity and polypragmasy. Multimorbidity is often associated with the use of multiple drugs in a single patient. This situation is called polypragmasia. Depending on the number of drugs taken, a distinction is made between polypragmasia (≥5 drugs) and severe polypragmasia (≥10 drugs) (7). This poses a risk of the so-called iatrogenic geriatric syndrome, which is a disease syndrome resulting from treatment. The use of multiple drugs exacerbates adverse drug effects, causes drug-drug interactions, causes drug-disease interactions, exacerbates the effects of other drugs (synergistic effects) or reduces the effectiveness of treatment (7, 8).

Falls are the leading cause of death among accident-related deaths in the over-65 age group (7). Still an underestimated consequence is the so-called post-fall syndrome, which is characterized by the fact that the person who has suffered a fall begins to limit their activity, fearing the further ones. Their performance deteriorates and, paradoxically, the risk of another fall is even higher (7). Bed immobilization of seniors promotes social isolation and significantly increases the risk of thromboembolic complications, bedsores and flares, chronic respiratory and urinary infections, and poses a risk of developing depression (2, 9, 10). In contrast, the fall itself is a factor that promotes the development of anxiety disorders.

Anxiety disorders are the most prevalent mental health problems among older adults (11). A review of the literature indicates that the prevalence of anxiety disorders ranges from 1.2 to 28% (12, 13). However, attention is drawn to the large discrepancies in epidemiological data, which are due to diagnostic difficulties and inadequate psychiatric care for older people. The most common anxiety disorders among geriatric patients include generalized anxiety disorder and specific phobias (11, 12). A study by Canuto et al. (13), which examined 3,142 people aged 65–84 years, found that the most common type of anxiety was agoraphobia (4.9%), paroxysmal anxiety disorder (3.8%), animal phobia (3.5%), post-traumatic stress disorder (1.4%) and social phobia (1.3%). The prevalence of the obsessive-compulsive disorder in this study was determined to be 0.8%. Women are particularly at risk of developing anxiety disorders, being diagnosed twice as often as men, and people between 65 and 75 years of age (13). Studies show that after the age of 75, the risk of developing an anxiety disorder decreases below 40%, and below 47% after the age of 80 (13).

In addition to the previously mentioned anxiety disorders, anxiety in geriatric patients (less frequently in children) is associated with fear of walking (ambulophobia) and fear of falling (basiphobia) (4, 14). People affected by these fears withdraw from daily activities in fear of falling and potential injury and its consequences. They are afraid to walk on flat, uneven surfaces, on unfamiliar terrain away from home or even in their place of residence (4, 6). This phobia may arise as a result of a traumatic experience, a past experience—the affected person may have experienced a fall or witnessed another person fall. As a consequence of this incident, they experience a real, but irrational and disproportionate fear of walking due to lack of confidence in their own abilities. This is the consequence of anticipating possible incidents and their effects. For example, a person suffering from dizziness and vestibular problems may fear falling and suffering a head injury.

Ambulophobia is caused by a well-defined factor and therefore constitutes a type of specific phobia. According to the Diagnostic and Statistical Manual of Mental Disorders, fifth edition (15), the following diagnostic criteria must be fulfilled for the diagnosis of specific phobia:

- Severe fear or anxiety about a specific object or situation;
- The fearful object or phobic situation always produces immediate fear or anxiety;
- The patient avoids the fearful object or phobic situation, otherwise, there is severe fear or anxiety;
- The severity of the fear or anxiety is inappropriate to the level of risk posed by the specific object or situation and to the socio-cultural context
- The anxiety, distress and avoidance are sustained and usually last 6 months or longer
- Anxiety, distress and the need for avoidance cause impairment in social, occupational and other important areas of functioning
- The disorder cannot be better explained by the presence of symptoms of another mental disorder.

People suffering from phobia often try to defend themselves against anxiety in various ways—mostly they avoid what arouses unpleasant feelings in them and use the escape mechanism. Unfortunately, this leads to a significant reduction in life opportunities (16).

Ambulophobia occurring in geriatric patients, due to the risk of large and irreversible complications, represents a significant clinical and social problem requiring a multidisciplinary approach (2, 9). It is, therefore, necessary to spread knowledge on this aspect among health care professionals and to raise awareness about it.

The aim of this study was to determine the prevalence of ambulophobia among patients of long-term care facilities in Poland and to identify factors that increase the risk of this specific phobia in the study group.

## Materials and Methods

### Study Group

Patients from 16 long-term care facilities (health care centers and nursing homes) located in Poland were included in the study. The directors/managers of each facility gave their consent for their institution to participate in the study.

The survey was conducted between January and July 2021. Data collected from 379 patients were analyzed. The participating sample was representative in terms of age and gender for the general population of patients in long-term care facilities in Poland (*p* = 0.89 and *p* = 0.73 respectively). Participation in the study was voluntary. Respondents whose cognitive functioning allowed them to understand the purpose of the study gave their independent and informed consent to participate in the study. For patients diagnosed with cognitive impairment, dual consent was required: given by the patient themselves and by their carer or family.

The study design was approved by the directors of the institutions where the study was conducted. According to Polish law, this study was not a medical experiment, so it did not require the consent of the Bioethics Committee (Act of December 5, 1996, on the professions of physician and dentist) (i.e., Journal of Laws 2019, item 537). Nevertheless, all research standards were observed in the study. It complies with the provisions of the Declaration of Helsinki.

The main inclusion criteria were: the patient's status in the respective long-term care facility, consent to participate in the study, and maintenance of basic independent mobility skills. Severely ill patients whose condition required prolonged bed rest (terminal stage of cancer, sequelae of traffic accidents, impaired consciousness, acute infectious diseases, e.g., pneumonia, COVID-19) were excluded from the study. Considering preserved mobility as a necessary factor for ambulophobia, participants who could not move independently and were in a wheelchair for somatic reasons (strokes, spinal cord and limb injuries, frailty syndrome) were also excluded from the study. Nonetheless, the study analyzed data from 6 patients who used a wheelchair despite the absence of any somatic complaints that would have prevented independent mobility (these were people who used a wheelchair because of fear of walking independently). Those with acute positive symptoms associated with schizophrenia were also excluded from the study.

### Research Tool

A survey questionnaire was used in this study, which consisted of a metric section (information was collected on age, sex, number of medications taken per day, comorbidities, mode of mobility, history of falling, seeing another person fall in the past, the subjective scale of the feeling of anxiety about walking (scale from 0 to 10 points, according to Likert scale assumptions, where 0—no anxiety about walking, 1—minimal anxiety, 10—maximum anxiety) and screening tests. Among the screening tests used were:

- Mini-Mental State Examination (MMSE)—for screening assessment of cognitive functioning. MMSE was adjusted for age and number of years of education. Scores were interpreted according to the following standard (17):
  - Normal score: 30–27 points;
  - Cognitive impairment without dementia: 26–24 points;
  - Mild dementia: 23–19 points;
  - Moderate dementia: 18–11 points;
  - Severe dementia: 10–0 points.
- Geriatric Depression Scale, short-form (GDS-15)—The GDS is a screening tool for assessing the severity of depressive symptoms in elderly people. Due to its short form, it is recommended for both healthy and neurologically impaired people. The maximum score that can be obtained is 15 points. Points were interpreted according to the following scale (18):
  - 0–5 points—no depression,
  - 6–10 points—moderate depression,
  - 11–15 points—severe depression.
- Delirium Observation Screening Scale (DOS)—a scale shortened to 13 points used to assess clinical symptoms of delirium in patients at risk (acute illness, cognitive impairment). Points were interpreted according to the following scale (17, 18):
  - 0 points—no presence of symptoms of delirium as assessed by the DOS scale was found,
  - 1–2 points—a probability of developing symptoms of delirium not meeting the criteria of the DOS scale was found. The current risk of developing delirium in the future.
  - 3 and more points—the presence of symptoms of delirium fulfilling the criteria of the DOS scale was found.
- Activities of Daily Living (ADL)—a scale assessing the ability to perform basic life activities by the person subject to the examination. It allows for the assessment of the current functional status of the patient, including the ability to self-care and the need for assistance from others. The scale was interpreted according to the following scoring (19):
  - 6–5 points—fully preserved activities;
  - 4–3 points—moderate impairment;
  - 2–0 points—severe functional impairment.

These screening tests were chosen because they are standardized and commonly used among geriatric patients. Their use allowed the assessment of selected parameters and minimized the risk of error associated with the use of less common, non-standardized tests.

The diagnostic criteria for specific phobia according to the Diagnostic and Statistical Manual of Mental Disorders, Fifth Edition, were used to assess the occurrence of ambulophobia. These criteria are presented in detail in the introduction of this article.

### Statistical Analysis

The characteristics of the study group with particular reference to ambulophobia and including age, polypragmasia, degree of dementia and fear of falling are presented in this paper. The normality of distributions was assessed using the Shapiro Wilk test. The χ^2^ test was used to test the significance of differences. Non-parametric correlation tests: ϕ and V Cramer were used in the analysis of associations between the examined variables.

Results for which *p* < 0.05 were considered statistically significant. Statistical analysis was performed using Statistica 13.3 PL program (StatSoft Polska, Krakow, Poland).

## Results

### Characteristics of the Study Group

The study involved 379 patients, of whom 58.8% (*n* = 223) were women and 41.2% (*n* = 156) were men. The mean age was 81.3 ± 8.5 years, with the youngest patient being 65 years old and the oldest 100 years old. With regard to age categories, the largest group was patients between 71–80 years (*n* = 132; 34.8%) and 81–90 years (*n* = 120; 31.7%), while less numerous groups were: ≤ 70 years (*n* = 52; 13.7%) and 91–100 years (*n* = 75; 19.8%) (Table 1).

**Table 1:** Characteristics of the study group (including gender division).

**Variable**	**Total *n*; %**	**Sex**		***p*** -value^*^
		**Women**	**Men**	
		***n***; **%**	***n***; **%**	
		**223; 58.8%**	**156; 41.2%**	
**Age (years)**				
≤ 70	52; 13.7%	28; 12.5%	24; 15.4%	0.15^*^
71–80	132; 34.8%	70; 31.4%	62; 39.7%	
81–90	120; 31.7%	74; 33.2%	46; 29.5%	
91–100	75; 19.8%	51; 22.9%	24; 15.4%	
**Occurence of polypragmasia**				
No polypragmasia	13; 3.4%	8; 3.6%	5; 3.2%	0.93^*^
Polypragmasia	296; 78.1%	175; 78.5%	121; 77.6%	
Severe polypragmasia	70; 18.5%	40; 17.9%	30; 19.2%	
**Occurence of ambulophobia**				
No ambulophobia	265; 69.9%	139; 62.3%	126; 80.8%	< 0.0001^*^
Ambulophobia	114; 30.1%	84; 37.7%	30; 19.2%	
**The way of movement**				
Alone	174; 45.9%	97; 43.5%	77; 49.3%	< 0.0001^*^
With the help of a walker	150; 39.6%	78; 35.0%	72; 46.2%	
With the help of a walking stick/crutch	49; 12.9%	42; 18.8%	7; 4.5%	
With the help of a wheelchair	6; 1.6%	6; 2.7%	0	
**Abnormal gait (including unsteady gait, shuffling gait, forward leaning gait)**				
Yes	222; 58.6%	139; 62.3%	83; 53.2%	0.001^*^
No	157; 41.4%	84; 37.7%	73; 46.8%	
**Screening for cognitive status (MMSE scale)**				
Normal score	47; 12.4%	26; 11.7%	21; 13.4%	0.13^*^
Cognitive impairment without dementia	59; 15.6%	42; 18.8%	17; 10.9%	
Mild dementia	111; 29.3%	65; 29.1%	46; 29.5%	
Moderate dementia	126; 33.2%	66; 29.6%	60; 38.5%	
Severe dementia	36; 9.5%	24; 10.8%	12; 7.7%	
**Screening for depression (GDS)**				
No depression	205; 54.1%	103; 46.2%	102; 65.4%	0.0006^*^
Moderate depression	126; 33.2%	84; 37.7%	42; 26.9%	
Severe depression	48; 12.7%	36; 16.1%	12; 7.7%	
**Screening for delirium (DOS)**				
Absence of symptoms of delirium	169; 44.6%	102; 45.8%	67; 42.9%	0.001^*^
Delirium symptoms not fulfilling DOS criteria	126; 33.2%	85; 38.1%	41; 26.3%	
Delirium symptoms satisfying DOS criteria	84; 22.2%	36; 16.1%	48; 30.8%	
**Assessment of daily functioning (ADL scale)**				
Fit person	78; 20.6%	48; 21.5%	30; 19.2%	0.2^*^
Person with moderate disability	175; 46.2%	109; 48.9%	66; 42.3%	
Person substantially disabled	126; 33.2%	66; 29.6%	60; 38.5%	

* *χ^2^ test. Bold values indicate statistical significance*.

Polypragmasia was common in the study group—it affected 96.6% of patients (*n* = 366), with its severe form occurring in 18.5% of patients (*n* = 70). The prevalence of polypragmasia was similar in men and women (Table 1).

Ambulophobia was noted in 30.1% of the surveyed individuals (*n* = 114). Ambulophobia was significantly more common (*p* < 0.0001) in women (*n* = 84; 37.7%) than in men (*n* = 30; 19.2%) (Table 1).

The way the patients moved was varied. The majority of study participants −45.9% (*n* = 174)—moved independently, followed by those with the help of a walker (*n* = 150; 39.6%), while the fewest used a wheelchair (6; 1.6%). Significantly more women (*n* = 42; 18.8%) than men (*n* = 7; 4.5%) moved with the help of a cane/ orthopedic crutch while moving with the help of a walker was more common among men (*n* = 72; 46.2%) than women (*n* = 78; 35.0%) (*p* < 0.0001) (Table 1).

Analysis of the MMSE scale results showed that the study group was dominated by patients with moderate dementia (*n* = 126; 33.2%) and mild dementia (*n* = 111; 29.3%), while the least number of patients whose MMSE results indicated severe dementia (*n* = 36; 9.5%) (Table 1).

The assessment of the severity of depression symptoms revealed their abscence in more than half of the respondents (*n* = 205; 54.1%). The risk of depression was significantly higher among women than men—moderate and severe depression was noted in 37.7% (*n* = 84) and 16.1% (*n* = 36) of women and 26.9% (*n* = 42) and 7.7% (*n* = 12) of men respectively (Table 1).

In 44.6% of the subjects (*n* = 169) there were no symptoms of delirium. Symptoms of delirium fulfilling the criteria of the DOS scale were more frequent among men (*n* = 48; 30.8%) than women (N = 36; 16.1%), while the opposite trend was observed for symptoms of delirium not fulfilling the DOS criteria. The differences within sex were statistically significant (*p* < 0.001) (Table 1).

Based on the results of the ADL scale, it was found that physically fit patients represented only about 20% of all patients (*n* = 78), while moderately unfit patients predominated (*n* = 175; 46.2%) (Table 1).

Based on the statistical analysis, ambulophobia was most common in patients aged 71–80 years (*n* = 48; 42.11%) (χ^2^ = 7.9; df = 3; *p* = 0.048) and in the group of people moving with the help of a walker (*n* = 73; 64.0%) (χ^2^ = 62.8; df = 3; *p* < 0.0001). Differences (*p* = 0.04) were found between those with ambulophobia aged 71–80 years and those aged 91–100 years in terms of how they moved—patients with ambulophobia aged 91–100 years were more likely to move independently (*n* = 10; 43.5%) than those aged 71–80 years (*n* = 10; 20.1%).

**Figure 1:**
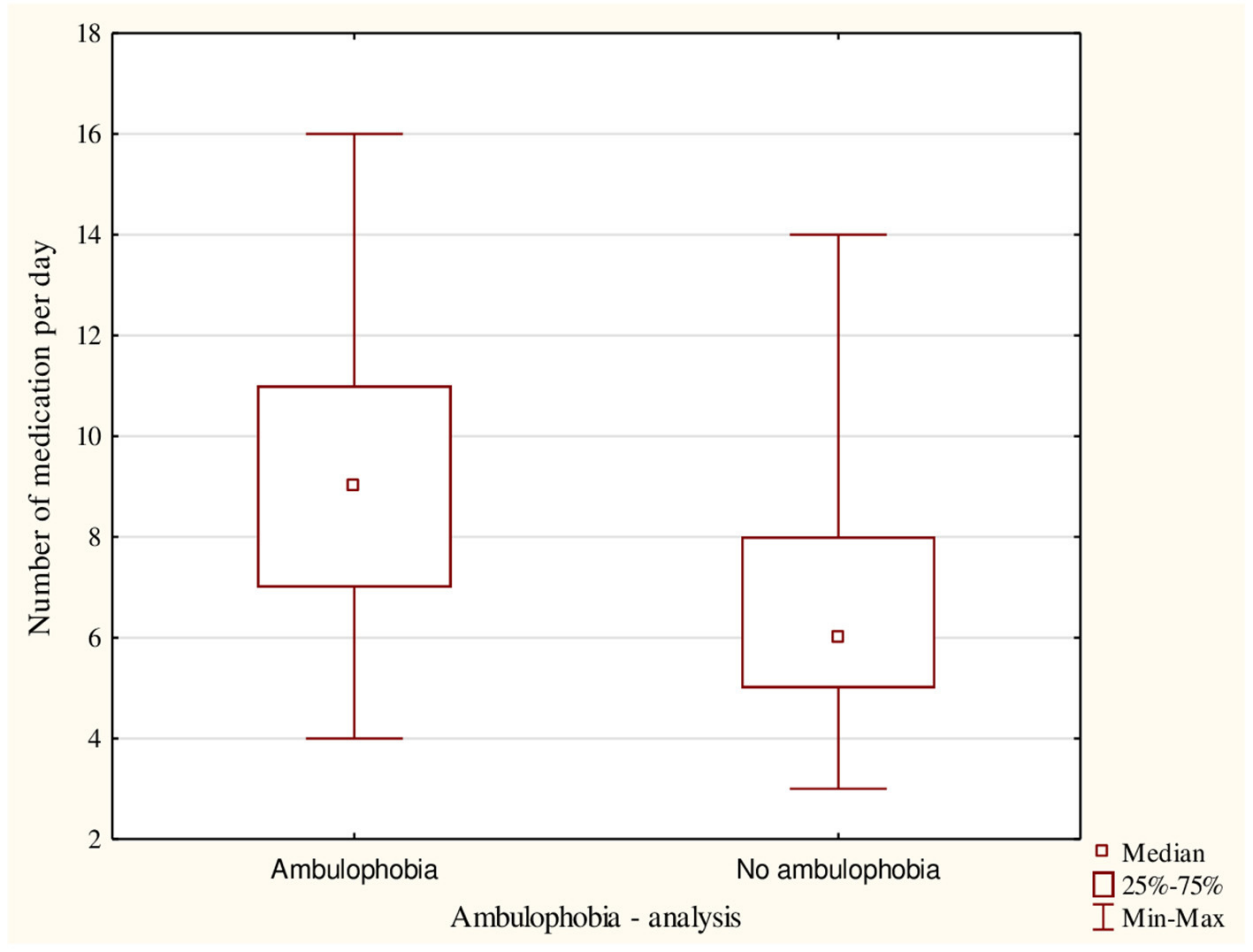
The number of medications taken among all participants including the presence of ambulophobia.

The presence of ambulophobia moderately correlated with the amount of medication taken (V = 0.47). It was observed, that patients with ambulophobia took more medication than those without diagnosed ambulophobia (Figure 1). Among the subjects with severe polypragmasia, those with ambulophobia (*n* = 45; 64.3%) were significantly more likely (χ^2^ = 51.0; df = 2; *p* < 0.0001) than those without ambulophobia (*n* = 25; 35.7%). Ambulophobia was not recorded among patients with no polypragmasia.

Ambulophobia was also associated with selected diseases—phobia of mobility was present in all patients diagnosed with Parkinson's disease (*n* = 48; 100%) (χ^2^ = 127.8; df = 1; *p* < 0.0001) and in 74.8% (*n* = 89) of those with depression (χ^2^ = 164.9; df = 1; *p* < 0.0001) (Table 2).

**Table 2:** Occurrence of ambulophobia among patients with diagnosed selected diseases.

**Disease entity**		**Ambulophobia**		***p*** value^*^
		**Yes**	**No**	
		***n***; **%**	***n***; **%**	
Parkinson's disease	Yes	48; 100%	0	***p*** < 0.0001^*^
	No	265; 80.1%	66; 19.9%	
Orthostatic hypotonia	Yes	16; 50%	16; 50%	***p*** = 0.01^*^
	No	98; 28.2%	249; 71.8%	
Alzheimer's disease	Yes	12; 33.3%	24; 66.7%	*p* = 0.6
	No	102; 29.7%	241; 70.3%	
Post-stroke condition	Yes	23; 29.1%	56; 70.9%	*p* = 0.8
	No	91; 30.3%	209; 69.7%	
Epilepsy	Yes	5; 29.4%	12; 70.6%	*p* = 0.9
	No	109; 30.1%	253; 69.9%	
Depression	Yes	89; 74.8%	30; 25.2%	***p*** < 0.0001^*^
	No	25; 9.6%	235; 90.4%	

* *χ^2^ test. Bold values indicate statistical significance*.

A correlation was also found between the results of selected tests used in comprehensive geriatric assessment and ambulophobia. About the MMSE scale results, it was observed that people with mild, moderate and severe dementia suffered less frequently from ambulophobia (χ^2^ = 51.0; df = 1; *p* < 0.0001) than those with normal results or those indicating the presence of cognitive impairment without dementia (Table 3). The strength of the correlation between the MMSE questionnaire results and the presence of ambulophobia can be described as average (V = 0.42).

**Table 3:** Occurrence of ambulophobia in the study group divided by the results of selected screening tests used in comprehensive geriatric assessment.

**Type of test and its interpretation**	**Ambulophobia**		***p*** -Value^*^
	**Yes**	**No**	
	***n***; **%**	***n***; **%**	
**Mini-Mental State Examination (MMSE)**			
Normal score	19; 40.4%	28; 59.6%	***p*** < 0.0001^*^
Cognitive impairment without dementia	39; 66.1%	20; 33.9%	
Mild dementia	23; 20.7%	88; 79.3%	
Moderate dementia	27; 21.4%	99; 78.6%	
Severe dementia	6; 16.7%	30; 88.3%	
**Geriatric Depression Scale (GDS)**			
No depression	24; 11.7%	181; 88.3%	***p*** < 0.0001^*^
Moderate depression	48; 38.1%	78; 61.9%	
Severe depression	42; 87.5%	6; 12.5%	
**Delirium Observation Screening Scale (DOS)**			
Absence of symptoms of delirium	60; 35.5%	109; 64.5%	***p*** = 0.0015^*^
Delirium symptoms not fulfilling DOS criteria	42; 33.3%	84; 66.7%	
Delirium symptoms satisfying DOS criteria	12; 14.3%	72; 85.7%	
**Activities of daily living scale (ADL)**			
Fit person	12; 15.4%	66; 84.6%	*p* = 0.006
Person with moderate disability	60; 34.3%	115; 65.7%	
Person substantially disabled	42; 33.3%	84; 66.7%	

* *χ^2^ test. Bold values indicate statistical significance*.

The GDS test results confirmed that ambulophobia was significantly more common among people with moderate and severe depression (χ^2^ = 112.0; df = 2; *p* < 0.0001). The greatest differences were noted in the case of severe depression—as many as 87.5% (*n* = 42) of people with severe depression met the criteria for ambulophobia (Table 3). The GDS scores strongly correlated with the presence of ambulophobia (V = 0.60).

The presence of symptoms of delirium, measured by the DOS scale, was also associated with ambulophobia. The greatest differences were observed in the case of delirium symptoms meeting the DOS criteria—among all subjects with delirium symptoms meeting the DOS criteria (*n* = 84), only 14.3% (*n* = 12) of the subjects had scores indicating ambulophobia (χ^2^ = 13.0; df = 2; *p* = 0.0015) (Table 3). A weak correlation was found between DOS questionnaire scores and ambulophobia (V = 0.22).

Statistical analysis of the correlation between ADL questionnaire scores and ambulophobia showed that the phobia of moving was least common among the able-bodied (*n* = 12; 15.4%). Furthermore, it was observed that any deterioration in the patient's physical fitness, interpreted on the ADL scale as moderate or severe disability, had a comparable impact on the occurrence of ambulophobia—the percentage of those with ambulophobia among the moderately unfit was 34.3% (*n* = 60) and for the severely unfit-−33.3% (*n* = 42). The observed differences were statistically significant (χ^2^ = 10.1; df = 2; *p* = 0.006) (Table 3). The association between the occurrence of ambulophobia and ADL questionnaire scores was defined as weak (V = 0.30).

**Figure 2:**
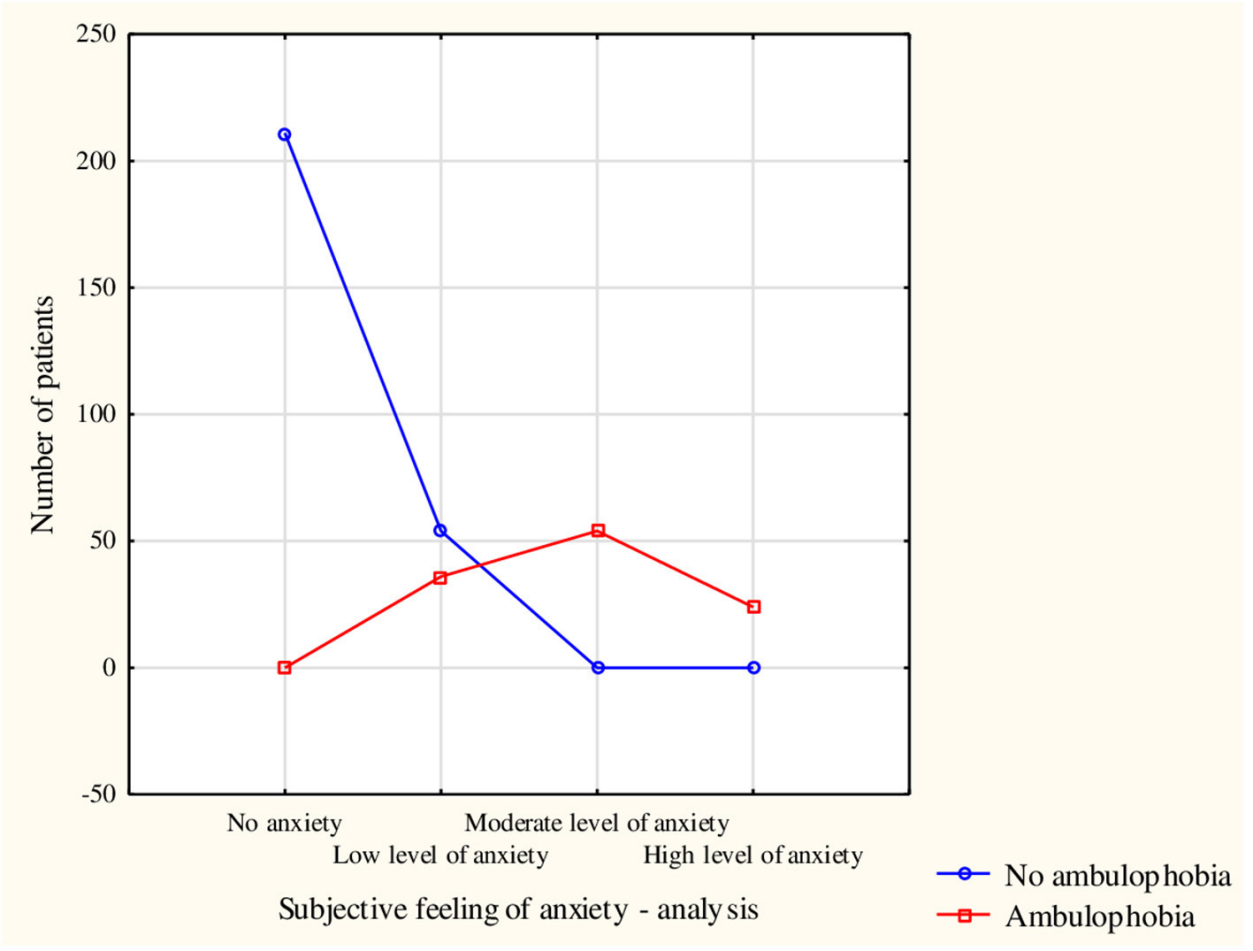
Occurrence of ambulophobia among respondents with a breakdown of subjective experience of fear of falling.

The subjective feeling of fear of falling had a significant effect on the perception of ambulophobia. Patients who did not experience anxiety also did not betray signs of ambulophobia, whereas subjects with high levels of fear of falling included only those characterized by ambulophobia (χ^2^ = 276.3; df = 3; *p* < 0.0001) (Figure 2). Based on Cramer's V coefficient (V = 0.88), a very strong correlation was found between the presence of ambulophobia and subjective feelings of anxiety.

The factor determining the more frequent occurrence of ambulophobia was an event in the form of a fall of a patient in the past or a fall of another person when the patient witnessed such a fall. In the entire study population, people with ambulophobia who had not experienced a fall accounted for 6.3% (*n* = 24), whereas in patients who had a fall, ambulophobia was almost twice more common, affecting 11.1% of the study population (*n* = 42) (χ^2^ = 42.8; df = 1; *p* < 0.0001). The strength of the correlation between the patient's own fall and ambulophobia was defined as moderate (ϕ = 0.34). The event of observing another person fall corresponded quite strongly with ambulophobia (ϕ = 0.53)—among patients who had seen a fall, the proportion with ambulophobia was as high as 17.4% (*n* = 66) (χ^2^ = 105.0; df = 1; *p* < 0.0001) (Table 4). Closer analysis showed that among those who had seen a fall, there was a full correlation between subjective feelings of anxiety and ambulophobia (V = 1.0), whereas, for those who had experienced a fall, the strength of the correlation was high (V = 0.7).

**Table 4:** Occurrence of ambulophobia in the total study population including falls experienced by the patient themselves or seen by them.

**Occurrence of ambulophobia in total study population including experience of own fall**			
**Ambulophobia**	**YES**	**NO**	***p*** value^*^
	***n***; **%**	***n***; **%**	
**Patient's fall**			
Yes	**42; 11.1%**	72; 19.0%	*p* < 0.0001^*^
No	24; 6.3%	241; 63.6%	
**Occurrence of ambulophobia in total study population taking into account the experience of watching another person fall**			
**Watching another person fall**			
	***n***; **%**	***n***; **%**	
Yes	**66; 17.4%**	48; 12.7%	*p* < 0.0001^*^
No	24; 6.3%	241; 63.6%	

* *χ^2^ test. Bold values indicate statistical significance*.

## Discussion

The rate of aging processes is characterized by multifactorial variation. The older people get, the more they differ from each other in their morphofunctional characteristics (20). In old age, the supply of energy substrates to muscle fibers is impaired due to a sparse capillary network (21). According to some authors, between the third and eighth decades of a person's life, the strength of the muscles of the lower limbs may decrease up to 40%, while that of the muscles of the upper limbs may decrease up to 30%. The reduction of static and dynamic strength of senior muscles has been confirmed (21).

Involutionary processes affect on the impairment of the functions of the motor and postural system, on which postural stability depends. Postural stability is understood as the resistance of the posture to endogenous and exogenous disturbances, the source of which can be both environmental variability and the interaction of the organism with the environment (20). Changes in gait pattern (unsteady, small step without detaching the sole from the ground), cardiovascular and respiratory conditions, labile compensatory mechanism during locomotion in the environment are further risk factors for falls (22). The experience of falling, on the other hand, poses a significant risk of developing an anxiety disorder in the form of a specific phobia—ambulophobia. It can affect a person in two ways. The first, positive, increases awareness which leads to more cautious behavior and positive fall prevention strategies. The second, negative, leads to avoidance of activities, which translates into social isolation, reduced functional ability and weakness, which can consequently exacerbate anxiety (23).

Despite the significant impact of ambulophobia on the daily functioning of older adults, the literature does not provide information on the epidemiology of that phenomenon and the factors that contribute to its occurrence. Instead, limited studies have investigated the associations between fear of walking and gait and musculoskeletal changes (21, 23).

Some studies (24, 25) have argued that fear of falling is common among older people, especially women, and is associated with a history of falls, gait and balance disturbances, depression, poor health, polypragmasia and reduced social activity. Similar results were obtained in our study. It was shown that ambulophobia was recorded in 30.1% of the individuals studied and was more than twice as common in women than in men. The epidemiology of this disorder is comparable to the study of Holtzer et al. (25). In the cited study, the prevalence of ambulophobia was determined to be 25.3%; however, in our work, many more patients were studied and the subjects had a higher mean age, which may have played a role in these small differences in values.

Great importance was also attributed to multimorbidity, which often occurs in older people. Our study showed that ambulophobia was significantly more frequent among patients with orthostatic hypotonia, Parkinson's disease and depression. Orthostatic hypotonia is a condition in which there is a decrease of at least 20 mmHg in systolic and diastolic blood pressure values associated with a change in body position (14). Rapid hypotension can trigger stupor, dizziness and, as a result, an uncontrolled fall during dynamic changes in body position in seniors. Studies indicate that this situation affects about 17–20% of patients undergoing hospitalization and about 33% of seniors living alone in the community (7, 14). One suspects that it occurs much more frequently among patients of long-term care facilities, but, due to the lack of data, it is difficult to determine the exact scale of the problem.

The study found that all patients who had Parkinson's disease met the diagnostic criteria for ambulophobia. This is a surprising result and there is need for more accurate research. Nevertheless, the characteristic presentation of Parkinson's disease, which includes motor slowing, clumsiness in movements, slowed mental processes, impaired balance or difficulty performing simple tasks such as getting up from a chair or bed, may drastically affect the occurrence of fear of movement and fear of falling (26). It is also worth noting that the people with Parkinson's disease participating in the study had a long history of treatment for Parkinson's disease and the disease itself was described as severe, which may have influenced the results.

The co-occurrence of ambulophobia and depression are shown in our study is not surprising. Co-occurrence of depression and anxiety is frequently observed in clinical practice. Similar observations have also been reported in other scientific reports (11, 12, 27). Considerating old age and progressive dysfunction in the musculoskeletal area, difficulties in self-care and less satisfaction with independent mobility, this type of anxiety, although often irrational and inadequate to the threat, seems to be the most real for the sufferer.

Atkin et al. (28) showed that 6–15% of adverse drug reactions are associated with the use of two drugs simultaneously and interactions between them, with more drugs this value increases exponentially. It has also been shown that older people are 10 times more likely to have drug interactions than younger people. Scientific reviews show that the more drugs used, the higher the risk of falling (14). This situation therefore directly translates into increased anxiety. In our study, it was shown that the majority of the subjects have polypragmasia or severe polypragmasia, and it was also shown that ambulophobia is more common in this group of respondents. These correlations can also be linked to the experience of falling oneself or witnessing another person fall.

The emotional state of a human being is a very complex aspect and depends on many factors. One element of emotional state is fear or anxiety. Fear is described as a state of strong emotional tension, appearing in situations of real danger. It is the desired state, connected with primal instincts conditioning survival in surprising and difficult situations. Anxiety, on the other hand, often takes a maladaptive form, disturbing human functioning. When it becomes dominant in behavior and prevents normal functioning, it becomes a pathology requiring treatment (29). The development of anxiety disorders can occur as a result of unmet emotional needs in childhood, experiencing difficult life experiences, feeling insecure, experiencing chronic stress or trauma. Any situation that negatively affects the mental state can also be the cause for the development of anxiety disorders (13). Such experiences include falling yourself or being a witness to another person falling. This is undeniably an unpleasant experience that can affect the senior's further life. A so-called “vicious circle” mechanism is created here. Anxiety arises as a result of the fall, which causes a refusal to move around independently and a kind of self-mobilization, which promotes a decrease in the person's independence and the emergence of dependence on a carer. These limitations cause a decrease in muscle strength, which in turn increases the risk of another fall and intensifies anxiety.

Furthermore, the feeling of this anxiety may increase as a result of experiencing an injury from a previous fall or witnessing another person's injury. Our study also showed that subjective feelings of fear of mobility were higher in those who met diagnostic criteria for ambulophobia than in those who did not, demonstrating the need for a standard assessment of mobility anxiety in every patient in a long-term care facility or geriatric ward. We also suggest that it should become standard practice to talk to a psychologist or psychogeriatrician after experiencing one's fall or witnessing another person's fall. Such early specialist consultation could prevent the development of full-blown ambulophobia and act as a preventative measure. It would also allow us to know the extent of the problem and thus increase the awareness of health care professionals and develop appropriate and effective treatments.

Research (30–35) show that various mental disorders are increasingly being diagnosed among older people. Despite this, practitioner-clinicians still face diagnostic difficulties for different diseases, which is related to the masking of symptoms and their different specificity in older people compared to younger patients. There are many reports in the literature (13, 29–32) referring to depressive disorders and generalized anxiety disorders among older people, but there is still a noticeable lack of publications on the prevalence of specific phobias, including ambulophobia, and as shown in our study this is a significant problem among geriatric patients. This paper provides a basis for further research and discussion on this topic.

We consider the main advantage of our publication to be its innovative character and the fact that it is the largest ever, multi-center, population-based, representative study of patients treated in long-term care facilities in Poland. As an advantage, we also point to the identification of many factors, which strongly correlate with ambulophobia, which gives grounds for special observation of these groups of patients and early specialist intervention. The selection of the group (patients of long-term care facilities) in our opinion is a strong point of this study, because in Polish and international publications, this group of patients is often neglected, and taking into account the fact of aging society more and more people will benefit from this form of care. On the other hand, such selection of the group may be a limitation of this study, because its results are not adequate for the whole population of Polish seniors. Therefore, the next stage of knowledge development in this area should be a study conducted among elderly people staying at home and treated on an outpatient basis rather than in the long-term care system. We are also aware of other limitations, which include the lack of analyses between ambulophobia and the level of education or the type of injuries sustained during a fall. It would also be important to research the correlation between ambulophobia and specific medications taken by patients. Unfortunately, given the scale of the study and the number of variables analyzed, this was not possible in this part of the publication.

As practitioners, psychogeriatricians, we believe that the topic of fear of movement and fear of falling is an important aspect of our professional work, encountered frequently in our practice, and we encourage further research on this topic. It is worth remembering that falls and accompanying injuries cause not only a health burden but also a socio-economic burden, so prevention of their occurrence seems to be particularly beneficial, not only for individual reasons but also from the point of view of society as a whole. Undiagnosed and untreated anxiety disorders, apart from being a major inconvenience, a sense of constant emotional tension and psychological discomfort, can lead to insomnia, somatic deterioration, addictions and increase the risk of suicide, which translates into a worsening of patients' quality of life.

In conclusion, the prevalence of ambulophobia in the study group was 30.1%. Ambulophobia significantly more often concerned women and people in the age group of 71–80 years. Depression, Parkinson's disease, orthostatic hypotonia, a history of falling or seeing another person fall, and moderate or severe disability in activities of daily living increase the likelihood of ambulophobia. This type of specific phobia is also significantly more common among patients with polypragmasia, which supports the need to evaluate the medications used and reduce them. On the other hand, protective factors against the development of ambulophobia include male gender, younger age, fewer medications used per day, and no previous falls or seeing another person fall.

## Data Availability Statement

The data that support the findings of this study are available from the corresponding author upon reasonable request.

## Ethics Statement

Ethical review and approval was not required for the study on human participants in accordance with the local legislation and institutional requirements. The patients/participants provided their written informed consent to participate in this study.

## Author Contributions

MGó led the conception of work and analyses, as well as the interpretation of data for publication and the writing of the manuscript. KK, KG, KB, MGr, JF, and BC contributed to the design and the writing of the manuscript, namely preparation and critical revision. MB supervised the analyses. RP was the primary reviewer of the paper and contributed many of the final revisions. All authors have read and approved the final version for publication.

## Funding

Upon acceptance for publication, the publication will be paid for by the Medical University of Silesia in Katowice.

## Conflict of Interest

The authors declare that the research was conducted in the absence of any commercial or financial relationships that could be construed as a potential conflict of interest.

## Publisher's Note

All claims expressed in this article are solely those of the authors and do not necessarily represent those of their affiliated organizations, or those of the publisher, the editors and the reviewers. Any product that may be evaluated in this article, or claim that may be made by its manufacturer, is not guaranteed or endorsed by the publisher.

## Abbreviations

ADL: activities of daily living
DOS: Delirium Observation Screening Scale
DSM-5: Diagnostic and Statistical Manual of Mental Disorders, fifth edition
GDS-15: Geriatric Depression Scale, short-form
MMSE: Mini-Mental State Examination
WHO: World Health Organization.
